# Miscellaneous

**Published:** 1888-03

**Authors:** 


### MISCELLANEOUS.

Mental Telegraphing by Red Men.—It is said that the Indians on the plains have always practiced a system of mental telegraphing among themselves, by means of which they communicate with each other almost instantaneously, and without messengers or signals. This mental telegraphing is by no means peculiar to the Indians on the plains of the United States. The same thing has been done by many people on the plains and among the mountains, both of America and other countries, and is to-day, and always has been, one method of manifesting knowledge known to and practiced by many persons.

The manner in which such communications are made seems to be, and is, a great mystery. Many theories about it have been suggested, all of which fall far short of satisfying the minds of people as to how it is done. The fact that such communications are sent and received, and that they are often genuine and true, and that such is one mode of manifesting knowledge, is now almost universally conceded.—*Missouri Republican.*

"Regulate the regulator." With pure blood comes good health. Use Warner's Log Cabin Sarsaparilla and secure both. Best remedy. Largest bottle. One hundred and twenty doses for $1.00.

Fall River's Haunted Rectory.—A midnight racket has disturbed the occupants of the Rock street rectory for nearly three weeks and has completely baffled all attempts to explain it from natural causes. New features are developed almost nightly. The rector, who has heretofore held that the trouble could be explained from natural causes, was finally led to try whether the servant girls were responsible for the phenomena, as is alleged by some, who believe one or both to be possessed of spiritualistic powers. He decided to send one of the girls away for a few days, and said that if the noises still continued he would send the other away also. Then if the trouble continued it would show that the girls were not responsible. One of the girls is now away and the trouble is as bad and the mystery as deep as ever. The noises which occur principally at night, consist of rappings, the continuous slamming of folding doors, sounds as of marbles rolling down stairs, and other noises equally strong. In addition to this, lamps are mysteriously lighted in different parts of the house, and rugs and bric-a-brac are disturbed during the night.

Paper Bottles.—Mr. L. H. Thomas, of Chicago, is the inventor and introducer of a novel industry which is nothing less than the preparation of paper bottles. The industry is at present chiefly confined to Chicago, but is spreading rapidly to other parts of the union and abroad. The bottles, it is perhaps needless to say, are unbreakable and are of various shapes and sizes. Special machinery is used in this manufacture. A sheet of paper glued on one side is, rolled into a tube of the desired diameter, which is then cut into various lengths. The tops and bottoms are then glued in and necks provided. The interior of the bottles is then lined with a flint composition which quickly hardens, and it is said that it will resist acids and spirits.

SNAKE STORIES,—One of the most amusing superstitions regarding the serpent tribe is the widespread belief that certain snakes have the power of breaking themselves into segments, and afterwards reuniting again. A species common to the western part of the country, known as the glass snake, is especially credited with this power. One of these animals was recently dissected at the Central Park Museum in New York, and was found to differ in no respect from other snakes, except that the tail was unusually long in proportion to the body. It is quite possible that the vertebral segments of the tail might be broken off by a blow, but under no circumstances could the several parts come together again. In referring to this matter, a correspondent of the *Scientific American* relates that on a certain occasion an earwig was cut into two pieces, and the separate parts placed on a table. The head part made a journey around the table, and then "backing up" to the posterior segment, joined itself together, and moved off as if nothing had happened. This entertaining tale is given soberly, as an actual fact; but we think it will be safe to let our readers judge of the truth of the story for themselves.

RATTLESNAKE BITES.—Eighty years ago Joseph Geer, the first settler at Long Eddy, N. Y., learned the cure from John Johnson, a half-breed Delaware Indian, who had his wigwam on the Pennsylvania side of the river at the foot of Long Eddy, and eked out a miserable existence hunting, fishing and supplying the settlers with lead from a mine somewhere in the vicinity, to which he would go and get a load and return the same day. Johnson, like most Indians, was an inveterate lover of whisky, and for a pint of it would let a rattlesnake bite him and then cure himself with his remedy, which, however, he would not reveal.

Geer always kept liquor in his house, and on an occasion when Johnson was recovering from one of his frequent spells of drinking to excess, and was suffering terribly for the want of liquor, Geer, by promising never to reveal the secret while Johnson was alive, succeeded in getting the remedy for a pint of whisky. A few years thereafter Johnson went off with a strolling band of Indians and never returned. Geer kept the remedy a secret, however, till from old age he was unable to answer the calls of the settlers when any of them or their stock had been bitten, and then told it freely to all. It is as follows:

Apply to the wound a poultice one-half each of common salt and indigo, mixed with cold water, and renew every two hours. Eat freely of the leaves, or drink often of a tea made from them, of a variety of the blue violet (V. Sagittata), commonly known as the "arrow-leaved" violet. If the bite be upon the leg or an arm, bind the leaves in a circle around it above and just beyond the swelling. Moisten with cold water as often as they get dry from the fever created by the poison, and renew two or three times a day.

During the time this remedy has been in use in Mr. Geer's neighborhood it has effected at least twenty cures upon human beings, a great many more upon beasts, and has never failed with either.—*Reading Times.*

---

### EPITAPH ON A UNIVERSALIST CLERGYMAN.

Here lies a shepherd of the fold,
Body and limb;
Because *he* wouldn't damn the world,
The world damned *him*.

